# Different biokinetics of nanomedicines linking to their toxicity; an overview

**DOI:** 10.1186/2008-2231-21-14

**Published:** 2013-02-22

**Authors:** Sara Mostafalou, Hamidreza Mohammadi, Ali Ramazani, Mohammad Abdollahi

**Affiliations:** 1 Faculty of Pharmacy and Pharmaceutical Sciences Research Center, Tehran University of Medical Sciences, Tehran, Iran; 2 Department of Clinical Toxicology, Baharlou Hospital, Tehran University of Medical Sciences, Tehran, Iran; 3 Faculty of Georesources and Materials Engineering, RWTH-Aachen University, Aachen, Germany

**Keywords:** Nanomedicine, Biokinetics, Nanotoxicology, Review

## Abstract

In spite of the extreme rise to the knowledge of nanotechnology in pharmaceutical sciences, there are currently limited experimental works studying the interactions between nanoparticles (NPs) and the biological system. Adjustment of size and surface area plays the main role in the reaction between NPs and cells leading to their increased entrance into cells through skin, gastrointestinal and respiratory system. Moreover, change in physicochemical reactivity of NPs causes them to interact with circulatory and cellular proteins differentially leading to the altered parameters of their biokinetics, including adsorption, distribution, translocation, transformation, and elimination. A direct relationship between the surface area, reactive oxygen species generating capability, and proinflammatory effects of NPs have been found in respiratory tract toxicity. Additionally, complement-mediated hypersensitivity reactions to liposomes and other lipid-based nanodrugs have been well defined. Inhalation studies of some NPs have confirmed the translocation of inhaled materials to extra pulmonary organs such as central nervous system (CNS) via olfactory neurons and induction of inflammatory response. Injectable uncoated NPs have a tendency to remain on the injection site while the poly ethanol glycol (PEG)-coated NPs can be notably drained from the injection site to get as far as the lymph nodes where they accumulate. This confirms the existence of channels within the extracellular matrix for NPs to move along. Furthermore, induction of DNA strand breaks and formation of micronuclei have been recorded for exposure to some NPs such as single-walled carbon nanotubes.

In the recent years, most of the studies have simply outlined better efficacy of nanodrugs, but few discussed their possible toxic reactions specially if used chronically. Therefore, we emphasize that this part of the nanoscience must not be undermined and toxicologists must be sensitive to set up suitable in vivo or in vitro toxicity models. A system for collecting data about the relationships between NPs’ structure-size-efficacy-toxicity (SSET) should be specified with special regard to portal of entry and target organ.

## Introduction

Nanotechnology is a multidisciplinary area, which utilizes knowledge from the fields of physics, chemistry, biology, medicine, material's science and engineering. New perspectives in medical sciences have been developed by the growth of nanotechnology in the last few decades so that nanoparticles (NPs) are expected to hold a key position in various parts such as diagnosis, imaging, and particularly, drug delivery. Even though, the biotechnology has made different potent pharmaceutics, some of these products face problems in biological systems. Modern nanotechnology provides new methods to achieve temporal and spatial site-specific delivery [1].

The term of NP is defined with regard to the size of particle. For material scientists, NPs are with the size of ~100 nm while for atmospherical scientists, the nucleation mode (<10 nm) of the atmospherical multimodal size distribution is often referred to NP. Furthermore, medical scientists define all particles below 1000 nm as NP. The fact that information on size characteristics is not always provided in publications causes confusion while search for NPs in published works is done. However, the particles with the sizes smaller than 100 nm is suggested to be considered as NPs in pharmaceutical studies [2].

NPs show unique physicochemical characteristics depending upon their chemical composition (purity, crystalline and electronic properties), small size (surface area and size distribution), surface structure (surface reactivity, surface groups, inorganic and organic coatings), solubility, shape, and aggregation. In the biological system, these properties of NPs have a significant impact on their cellular uptake, protein binding, translocation from way of entry into the target site and the possibility of tissue injury so that concerns over their adverse effects have been raised recently [3]. For instance, complement-mediated hypersensitivity reactions to liposomes and other lipid-based nanodrugs by the classical or the mannose-binding lectin pathway or the alternative pathway have been recognized that necessitates special attention of toxicologists to emerge establishment of specific toxicity tests [4].

In the present article, we have focused on the kinetics of NPs and their potential interactions with fluids, cells, tissues via a range of possible pathways towards target organs. All nanomedicinal products must undergo special safety tests in order to ensure they do not cause any potential hazard. Although, environmental accumulation and degradation of these compounds are important to be evaluated, that is beyond the scope of the present review. To avoid testing in several species of animals, information gathered on metabolism, pharmacokinetics, and toxicology would end up in cost benefit tests.

### Biokinetics of NPs

The main characteristic of NPs is their size, which can modify the physicochemical properties of the material and create a different biokinetics which can lead to occurrence of toxic effects at nontoxic doses. In this way, understanding biokinetics of NPs can provide information on the amount of doses reaching body organs, in comparison with the non-NP form of own materials. Furthermore, NPs’ biokinetics is important for manipulating *in vitro* studies since it helps to determine sensitive target organs and explain the selection of NP concentrations in cell culture media, while studying effects and mechanisms in cells of a specific target organ. According to previous studies, biokinetics and toxicity of NPs depend on particle size, aggregation shape, chemical composition or crystal structure, surface area, surface chemistry, surface charge, surface composition, stability, solubility, and the rate of material release during dissolution [5].

From a toxicological attitude, particle size and surface area are important characteristics for NPs. Smaller size can create the opportunity for increased uptake and interaction with different parts of biological system. In the circulation, NPs are mainly taken up by mononuclear phagocytic cells acting as a depot and defense mechanism. The rate of this uptake is a determinant factor for the half-life of NPs in the blood. Some circulatory proteins like opsonins bind NPs in the blood and increase the rate of phagocytosis. In fact, opsonization assists the NPs to attach the superficial part of phagocytic cells, particularly monocytes, macrophages, neutrophils and dendritic cells via particular receptors. Solubility is a key factor in opsonization so that hydrophobic NPs tend to be easily attached rather than hydrophilic and neutral ones. Dissimilar to this condition, coating with hydrophilic polymers such as polyethylene glycol (PEG) counteracts with opsonization, which permits NPs to stay in the circulation for much longer periods and carried throughout the body to reach most of the tissues and cells [6].

Vascular endothelium with tight junctions is the next barrier for passage of NPs into the cells. Brain endothelial junctions are mainly close-fitting and effectual but in some specialized tissue such as liver, the endothelium is perforated and lets particles up to 100 nm to pass and go in the critical parenchymal cells. In spleen, the endothelium is inconsistent and contains greater fenestrations even without basement membrane, which allows very large particles to pass. Of course, in certain inflammatory conditions, the endothelium becomes more permeable even to bigger particles. Still, particles can pass through the cells by pinocytosis. Although the small size may be favorable for a rapid entry into the cells, no size cut off limit up to at least 5 μm can be estimated for pinocytosis. A major problem in dismembering the special effects of the size is high polydispersity of various NPs that seems dependent on the chemical structure [7].

Distribution of NPs into the organs is mainly done in liver followed by spleen, lymph node, and bone marrow. Some NPs can cross blood–brain barrier (BBB), an uptake which can be more facilitated by negative surface charge and binding to proteins such as albumin, Apo, and immunoglobulin G. NPs are found to be eliminated via urine or bile excretion in unmetabolized form, though there is some evidence on their metabolism [5]. Extra entrance through BBB not only is undesirable in some cases but may initiate serious side effects and toxicity.

However, the interaction between NPs and proteins plays the main role in their connections, distribution, transformation, accumulation, and clearance in the body. These interactions are mainly related to the corporeal form and chemical composition of NPs. For instance, implicating on scavenger-related elimination; NPs are likely to have a restricted distribution, while it has been evident that physical and chemical changes in NPs’ nature considerably influence their distribution [8].

Furthermore, decreased size and increased surface area can lead to the more atoms or molecules to be exposed to the surface rather than the interior of the material. In fact, the number of atoms or molecules at the surface of the particle can define the material reactivity and biological efficiency [9] which in turn might result in unanticipated toxicities.

### Altered biokinetics as a source of NPs toxicity

The result of some studies have shown that change in physicochemical properties of NPs due to their decreased size and increased surface area can create a series of toxic effects. For instance, in the lung, a direct relationship between the surface area, reactive oxygen species (ROS)-generating capability, and proinflammatory effects of NPs has been found. Additionally, studies show that the small size and large surface area of NPs increase their ability to induce lung injury [9].

Pulmonary and cardiovascular effects have been mostly attributed to inhalation exposure to smaller particles. Nevertheless, the evidence on how well particles can cross particular tissues outside the circulation is more sparse. Inhalation studies of some NPs have confirmed the translocation of inhaled materials to extra pulmonary organs. It has been shown that following nasal application, inhaled NPs can even get access to the central nervous system (CNS) via olfactory neurons and substantially fire the inflammatory response [10].

A comparison between some effects of NPs (<100 nm) and larger particles (>500 nm), when they are taken up via the respiratory tract was described by Oberdörster [11]. Regarding respiratory tract as portal of entry, it was recognized that “translocation to secondary organs, lymphatic circulation, uptake and transport by sensory neurons, protein/lipid adsorption, entry to cells, entry to mitochondria, entry to nucleus, direct effect on the portal of entry and on the secondary organs” are among the characteristics that seen in NPs smaller than 100 nm whereas particles larger than 500 nm, only produced direct effect on the portal of entry.

The injected NPs into the extracellular matrix can get back to the circulation by drainage of lymphatic vessels and lymph nodes [12]. Although uncoated NPs have a tendency to remain near the injection site, the poly ethanol glycol (PEG) coated NPs can be conspiciously drained away from the injection site. Coating’s thickness is an important factor as thin coatings cause NPs to get as far as the lymph nodes where they can accumulate. In thicker coating, they can depart from the lymph nodes and get back to the circulation. This supports the idea of existence of channels within the extracellular matrix for NPs to move along [13]. Once and for all, induction of DNA strand breaks and formation of micronuclei have been preserved for exposure to some NPs such as single-walled carbon nanotubes [14].

## Conclusion

The link between specific kinetics and induction of toxicity is now a well-known fact and not hidden but feels undermined for nanodrugs. Apart from that, nanodrugs are able to pass through different cellular barriers much more than that of usual drugs; their biologic behavior is under influence of synergistic interaction between physical and chemical characteristics. In the recent years, most of the studies have simply outlined slightly better efficacy of nanodrugs, but few discussed their possible toxic reactions specially if used chronically. Therefore, we emphasize that this part of the nanoscience must be re-considered and toxicologists must be sensitive to set up suitable in vivo and in vitro toxicity models. This means that each new nanodrug must undergo full toxicity tests even if its non-nano form has been already used and found safe. In this respect, it is reasonable to mention that most of the toxicity studies for nanodrugs in the recent years have been conducted in vitro. The limitation of such in vitro models is that most of them are conducted on the neoplastic passaged cell lines. Such results are under question since the function and responses of such cell lines are not fully similar to that of normal fresh cells [15]. Therefore, for efficacy and safety evaluation of nanodrugs, a method based on an approved animal model or an appropriate primary normal cell culture is assertively recommended. This so important task should be paid proper attention to completely fulfill the criteria to prove safety of nanodrugs. Additionally, since some nanodrugs are currently in therapeutic use, collection of adverse reaction and toxicity reports from the community will help health professionals and regulatory organisations to decide about their safety and usage. A system for collecting data about the relationships between NPs’ structure-size-efficacy-toxicity (SSET) should be specified with special regard to portal of entry and target organ.

## Authors’ contribution

All authors have made substantive contributions to the paper and read and approved the final manuscript.
